# Study on the Structural Features of Eight *Dendrobium* Polysaccharides and Their Protective Effects on Gastric Mucosa

**DOI:** 10.3390/foods13183011

**Published:** 2024-09-23

**Authors:** Haonan Wang, Ying Wang, Yuanxi Liu, Jinxin Xie, Yazhong Zhang, Hongyu Jin, Feng Wei, Shuangcheng Ma

**Affiliations:** 1 State Key Laboratory of Drug Regulatory Science, National Institutes for Food and Drug Control, Beijing 102629, China; hanor312@163.com (H.W.); wayi_1986@163.com (Y.W.); liuyuanxi@nifdc.org.cn (Y.L.); xiejx@nifdc.org.cn (J.X.); jhyu@nifdc.org.cn (H.J.); 2 National Institutes for Food and Drug Control, Chinese Academy of Medical Sciences & Peking Union Medical College, Beijing 100050, China; 3 NMPA Key Laboratory for Quality Research and Evaluation of Traditional Chinese Medicine, Anhui Institutes for Food and Drug Control, Hefei 230051, China; 13956985695@139.com; 4 Chinese Pharmacopoeia Commission, Beijing 100061, China

**Keywords:** *Dendrobium*, polysaccharide, physicochemical characterization, gastric mucosal protection, *D. huoshanense*

## Abstract

This study aimed to analyze the structure of polysaccharides from eight different *Dendrobium* species and their protective effects on gastric mucosa. Ultraviolet (UV) analysis showed that the contents of eight polysaccharides ranged from 51.89 ± 6.91% to 80.57 ± 11.63%; the degree of acetylation ranged from 0.17 ± 0.03 to 0.48 ± 0.03. High-performance liquid chromatography (HPLC) results showed that these polysaccharides were mainly composed of mannose (Man) and glucose (Glc) with a small amount of galactose (Gal) and arabinose (Ara), and the monosaccharide ratios of different *Dendrobium* species were different. High-performance size exclusion chromatography—multi angle light scattering—refractive index detector (HPSEC-MALS-RID) showed that the molecular weight (Mw) of all *Dendrobium* polysaccharides was >1 × 10^5^ Da; *D. huoshanense* had the lowest molecular weight. Subsequently, an ethanol injured GES-1 cell model was constructed to evaluate the gastric mucosal protective potential of polysaccharides from eight different *Dendrobium* species. The results showed that the protective effect of the low concentration 50 μg/mL DHP treatment group was similar to that of the control group (*p* > 0.05), and the cell viability could reach 97.32% of that of the control group. Based on the polysaccharide composition, different kinds of *Dendrobium* have different degrees of migration and repair effects on GES-1 damaged cells, and the effect of DHP is slightly better than that of other varieties (83.13 ± 1.05%). Additionally, *Dendrobium* polysaccharides alleviated ethanol-induced oxidative stress and inflammatory response in gastric mucosal cells by enhancing the activity of antioxidant enzymes (superoxide dismutase, glutathione peroxidase, catalase) and reducing the levels of malondialdehyde and reactive oxygen species. Overall, DHP can most effectively protect gastric mucosa. These findings enhance our understanding of the relationship between the structure and biological activity of *Dendrobium* polysaccharides, providing a foundation for the quality control of *Dendrobium*. Furthermore, these findings offer theoretical support for the development of *Dendrobium* polysaccharides as nutraceuticals to treat digestive system diseases.

## 1. Introduction

*Dendrobium* plant has medicinal and edible properties. Its medicinal value can be traced back to *Shennong Materia Medica Classic*, which states, “Long-term consumption strengthens the stomach and intestines [1,2].” The fresh and dried stems of *Dendrobium* are regarded as precious species and are widely used in the production of healthcare products and herbal tea beverages in China and Southeast Asian countries [3,4]. The 2020 edition of the *Chinese Pharmacopoeia* lists five *Dendrobium* species: *Dendrobium* huoshanense Z. Z. Tang & S. J. Cheng, *Dendrobium officinale* Kimura & Migo, *Dendrobium nobile* Lindl, *Dendrobium chrysotoxum* Lindl, *Dendrobium fimbriatum* Hook [5]. *Dendrobium* contains a variety of chemical components, such as polysaccharides, alkaloids, phenolic compounds, flavonoids, and sesquiterpenes, that provide medicinal value to the species. Among them, polysaccharides are the main active components of *Dendrobium* and have attracted significant attention due to their potential medicinal value in exerting immunomodulatory, hepatoprotective, anti-inflammatory, antioxidant, anti-tumor, and gastrointestinal protection effects [6,7,8].

Currently, many species of *Dendrobium* are available on the market, of which *D. huoshanense* and *D. officinale* are frequently counterfeited due to their high cost. This phenomenon severely affects the sustainable development of *Dendrobium* industry. The variations in polysaccharide composition among different species of *Dendrobium* are likely to influence their pharmacological and pharmacodynamic effects. Nonetheless, a comprehensive and systematic analysis of the polysaccharide composition in *Dendrobium* species is lacking. Xu et al. [9] compared the structures of different species of *Dendrobium* polysaccharides using saccharide mapping. The results showed that different species of *Dendrobium* polysaccharide components have similar properties, albeit with significant differences. The varied impact of different types of *Dendrobium* polysaccharides on macrophage function [10] indicates the complex relationship between polysaccharide molecular structure and biological activity.

Notably, the main function of *Dendrobium*, “nourishing the stomach”, is consistent with the modern pharmacological understanding of its protective effect on gastric mucosa. According to the World Health Organization’s 2024 report, alcohol is identified as a major risk factor leading to gastric cancer, a fatal malignancy [11]. The global rise in alcohol consumption levels [12] and long-term or excessive drinking can cause acute or chronic damage to the gastric mucosa, thereby increasing the risk of gastric ulcers [13]. Currently, some studies have confirmed the gastric mucosal protective effect of *Dendrobium* polysaccharides [4,14,15,16]. For example, *D. officinale* leaf polysaccharide can prevent LPS-induced GES-1 cell injury by inhibiting the release of inflammatory cytokines regulated via the TLR4/NF-κB signal pathways [15]. *D. huoshanense* stem polysaccharide could strengthen the gastric mucosal barrier to inhibit oxidative stress and NF-κB-driven inflammation-induced gastric mucosal injury [16]. However, there is still a lack of in-depth comparative studies in this field. That is, there is no consensus on the type of *Dendrobium* polysaccharides with the better gastric mucosal protective effect. Hence, it is important to compare the potential of different types of *Dendrobium* polysaccharides in the prevention and treatment of ethanol-induced gastric mucosal damage protection.

**Figure 1:**
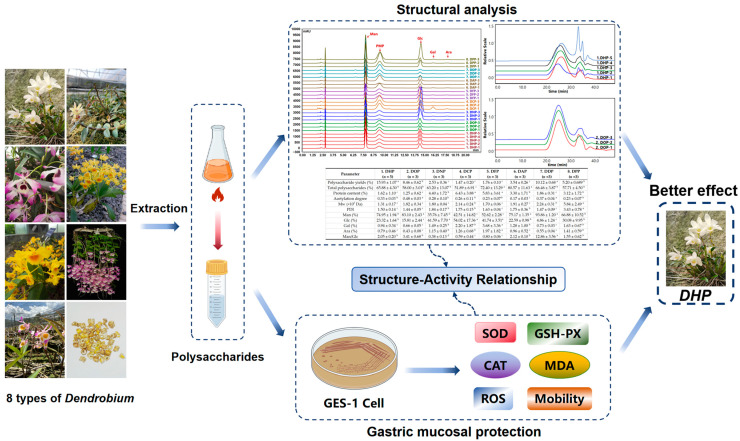
Graphical summary.

Therefore, the present study selected eight species of *Dendrobium*: *D. huoshanense*, *D. officinale*, *D. nobile*, *D. chrysotocum*, *D. fimbriatum*, *Dendrobium aphyllum* (Roxb.) C.E.C.Fisch, *Dendrobium devonianum* Paxton, and *Dendrobium pierardii* R.Br. The selection of these species was based on an in-depth study of the market and origin. We compared the structural differences between the various types of *Dendrobium polysaccharides* and investigated their efficacy in protecting the gastric mucosa (See Figure 1 for a summary of the detailed ideas). Our findings will provide a standard for the quality control of *Dendrobium* and a theoretical foundation for developing nutraceuticals and pharmaceuticals to protect gastric mucosa.

## 2. Materials and Methods

### 2.1. Materials and Reagents

Eight species of *Dendrobium*, including *D. huoshanense*, *D. officinale*, *D. nobile*, *D. chrysotocum*, *D. fimbriatum*, *D. aphyllum*, *D. devonianum* Paxton, and *D. pierardii*, were collected in 26 batches (*D. huoshanense*: five batches; others: three batches each), mainly from Anhui, Hubei, Zhejiang, and Yunnan provinces. The polysaccharides extracted from these species were named in the order *Dendrobium* polysaccharides (1–8): 1. DHP, 2. DOP, 3. DNP, 4. DCP, 5. DFP, 6. DAP, 7. DDP, and 8. DPP, respectively. Standard monosaccharide references, mannose (Man), glucose (Glc), and galactose (Gal), were obtained from the National Institute for Food and Drug Control (Beijing, China). Arabinose (Ara) and β-d-glucose pentaacetate were obtained from Shanghai Nature Standard Co., Ltd. (Shanghai, China). Phenyl-3-methyl-5-pyrazolone (PMP) was purchased from Sigma-Aldrich (St. Louis, MO, USA). Trifluoroacetic acid (TFA) and FeCl_3_ were procured from Oka Co., Ltd. (Beijing, China). Water was purified on a Millipore Milli-Q Plus system (Millipore, St. Louis, MO, USA).

GES-1 cells were obtained from Tianjin Hongshunke Biotechnology Co., Ltd. (Tianjin, China). Trypsin-EDTA, MTT Cell Proliferation and Cytotoxicity Assay Kit, and reactive oxygen species (ROS) Assay Kit were purchased from Beijing Solepol Science and Technology Co., Ltd. (Beijing, China). Catalase (CAT) Assay Kit and Malondialdehyde (MDA) Enzyme-Linked Immunosorbent Assay (ELISA) Kit were purchased from Wuhan Elite Bio-technology Co., Ltd. (Wuhan, China). Superoxide Dismutase (SOD) and Glutathione Peroxidase (GSH-Px) ELISA Kits were purchased from Wuhan Beinlai Biotechnology Co., Ltd. (Wuhan, China). Anti-ERK1/2, anti-JNK, and anti-p38 antibodies were purchased from Cell Signaling Technology Co., Ltd. (Boston, MA, USA). Bradford Protein Assay Kit, sodium dodecyl sulfate-polyacrylamide gel electrophoresis (SDS-PAGE), and Anti-GAPDH (glyceraldehyde-3-phosphate dehydrogenase) antibody were obtained from Boster (Wuhan, China). All the other chemicals and reagents used were of analytical grade.

### 2.2. Preparation of Polysaccharides from Different Cultivars of Dendrobium

The herbs were crushed into powder and passed through a no. 3 sieve according to the extraction method (water extraction and alcohol precipitation) of *Dendrobium* polysaccharides described in Chinese Pharmacopoeia [5]. Approximately 2 g of the powder was weighed precisely, mixed with 200 mL of water, heated, and refluxed for 2 h. The mixture was filtered and concentrated to about 20 mL, and then anhydrous ethanol was added to reach the concentration of 80% to precipitate the polysaccharides. The concentrate was incubated at 4 °C, for 12 h before centrifugation at 5000 rpm for 10 min at room temperature. The supernatant was discarded, and the precipitate was air-dried and solubilized in water.

In order to ensure the accuracy of the results of cellular experiments, we deproteinized the extracted *Dendrobium* polysaccharides to improve their purity using the Sevag method [17]. First, the proteins in the crude polysaccharides were removed by adding one-fifth volume of Sevag reagent (trichloromethane: *n*-butanol, 4:1, *v*/*v*), and shaken thoroughly with an incubator shaker at 250 rpm for 30 min. The supernatant was collected by centrifugation of the mixture at 5000 rpm for 10 min; the process was repeated several times until no denatured protein remained at the junction. The deproteinized polysaccharide extract was evaporated in a water bath to remove organic reagents and obtain the crude compound.

### 2.3. Structural Characterization of Dendrobium Polysaccharides

#### 2.3.1. Chemical Composition of the Polysaccharides

The carbohydrate content in the *Dendrobiums* was measured using the phenol-sulfuric acid method [18], whereas the protein content was analyzed by the Bradford method [19].

#### 2.3.2. Determination of Molecular Weight (Mw) and Polymer Dispersity Index (PDI)

The Mw distribution of *Dendrobium* polysaccharides was detected by high-performance size-exclusion chromatography—multi-angle light-scattering—refractive index detection (HPSEC–MALS–RID) method according to the method described by Fan et al. [20]. *Dendrobium* polysaccharide was resuspended in 5 mL of NaCl (0.1 mol/L) to achieve the final concentration of about 5 mg/mL; the mixture was filtered through 0.45 μm Millipore filter (Shanghai Anpu Co., Ltd., Shanghai, China) to remove impurities and subjected to chromatographic separation on a Shodex SB-806 gel column (300 mm × 8.0 mm, 13 μm) and a Shodex SB-804 gel column (300 mm × 8.0 mm, 10 μm) using the following parameters: the mobile phase consisted of 0.1 M NaCl aqueous solution at a flow rate of 0.6 mL/min for 50 min; the injection volume was 100 μL, and the column temperature was 40 °C. Data were collected and analyzed using Astra software (version 7.1.3, Wyatt Technology Co., Santa Barbara, CA, USA).

#### 2.3.3. Monosaccharide Composition Analysis

Each sample (2 mg/mL) was hydrolyzed with TFA (1 mol/L) at 120 °C for 4 h. The sample was blow-dried under nitrogen, mixed with 2 mL of methanol, and blow-dried again. This process was repeated three times to remove the remaining TFA; the pellet was resuspended in 1 mL of water. Then, 400 μL of 0.3 moL/L NaOH solution was added to the solution, and 400 μL of 0.5 moL/L PMP-methanol mixture was added with continued reaction at 70 °C for 100 min. After cooling to room temperature, 500 μL of 0.3 mol/L HCl was added to the mixture, followed by extraction (three times) with 3 mL of CH_3_Cl to remove the lower organic layer. The upper layer was retrieved and filtered through a 0.22-μm Millipore filter for high-performance liquid chromatography (HPLC) detection.

The separation was performed on an Agilent Zorbax SB-C18 column (4.6 mm × 250 mm, 5 μm) with acetonitrile and 0.02 mol/L ammonium acetate (20:80, *v*/*v*) as the mobile phase and the following parameters: flow rate 1.0 mL/min, column temperature 35 °C, sample size 10 μL, wavelength 250 nm, and duration 20 min. The monosaccharide standards were derivatized using the above method. The mixture of all monosaccharide standards, including Man, Gal, Glc, and Ara, was measured under identical chromatographic conditions, and the values were expressed as percentage molar ratios [4,21].

#### 2.3.4. Acetylation Assay

The degree of acetylation (DA) of *Dendrobium* polysaccharides was determined using a modified hydroxylamine colorimetric method [22]. FeCl_3_ served as the color developer, and β-d-glucose pentaacetate was used as the standard.

### 2.4. Cell Experiments

#### 2.4.1. Cell Model

GES-1 cells were inoculated in 96-well plates at a density of 8 × 10^3^ cells/well, with three replicate wells in each group, and cultured overnight in the incubator at 37 °C under 5% CO_2_. The cells were treated with different concentrations of ethanol to induce injury, and cell survival was determined after 0, 24, 48, and 72 h for further experiments. Next, the effects of eight groups of *Dendrobium* polysaccharides were investigated at different concentrations and time points on the viability of normal GES-1 cells. This approach determined the cytotoxicity and concentration-dependent effects of the *Dendrobium* polysaccharides on GES-1 cells and screened the most effective time and concentration of the polysaccharide treatment. For cellular experiments, representative batches of each *Dendrobium* species were screened, i.e., samples whose structural data were in the intermediate range were examined. Thus, the selected samples reflected the typical characteristics of the respective *Dendrobium* species, facilitating a broad application of the results of subsequent experiments. Then, to test the effect of eight species of *Dendrobium* polysaccharides on ethanol-induced cell damage, GES-1 cells were pretreated with different concentrations of the polysaccharides for 48 h. Except for the blank control group, all groups were treated with 1.0 mol/L ethanol for 4 h. After removing the supernatant, 90 μL of fresh culture medium was added, followed by 10 μL of MTT reagent to the cells. Finally, absorbance was measured on a microplate reader (Shanghai, China) at 490 nm. Cellular modeling aimed to determine the most effective concentration of *Dendrobium* polysaccharide for constructing an in vitro prophylactic model for GES-1 cells. 

#### 2.4.2. Grouping of Cells

GES-1 cells were grouped as follows: Control; Model (cells in 1.0 mol/L ethanol for 4 h); Experimental (after 48-h pretreatment with different concentrations of *Dendrobium* polysaccharides, cells were stimulated with 1.0 mol/L ethanol for 4 h). The experimental group consisted of eight species of *Dendrobium* polysaccharides, each at four different concentrations (25, 50, 100, and 250 μg/mL, respectively), resulting in a total of 32 subgroups.

#### 2.4.3. Cell Scratch Test

The cell scratch assay was used to assess the migratory ability of GES-1 cells. A marker was used to draw lines evenly on the back of the six-well plate, with each line about 0.5 cm apart. The cells were inoculated into six-well plates at a density of 1 × 10^5^ cells/well and incubated for monolayer formation. Then, the cell wound was scribed perpendicular to the labeled lines with a 20-mL pipette tip. The detached cells were washed three times with phosphate-buffered saline (PBS). Serum-free medium was added to the blank and model groups, and eight different *Dendrobium* polysaccharide solutions (50 μg/mL) were added to the experimental group. Each concentration group was set up in triplicate wells and incubated at 37 °C under 5% CO_2_. The healing of the scratched wounds was measured at 0, 24, and 48 h. The cell migration ability was measured using ImageJ (version 1.54 h, National Institutes of Health, Bethesda, MD, USA).

#### 2.4.4. Measurements of Oxidative Stress and Antioxidant Biomarkers via a Biochemical Approach

Cell samples were collected from the Control, Model, and Experimental groups (eight groups of *Dendrobium* polysaccharides, 50 μg/mL). After centrifugation at 1000 rpm for 10 min, the medium was discarded, and the cell pellet was rinsed three times with pre-cooled PBS. Subsequently, the pellet was resuspended in an appropriate volume of pre-cooled PBS and lysed by repeated freeze-thawing: three cycles of freezing at −20 °C or −80 °C for 30 min and thawed at 37 °C. The lysate was clarified by centrifugation at 10,000 rpm, 4 °C for 10 min. Finally, the supernatant was used to measure the activities and levels of SOD, CAT, GSH-Px, and MDA according to the manufacturer’s instructions.

#### 2.4.5. ROS Detection

Intracellular ROS levels were measured using 2,7-dichlorofluorescein-diacetate (DCFH-DA) and ROS Kit, according to the manufacturer’s instructions. Briefly, the grouped cells were collected, suspended in diluted 10 μmol/L DCFH-DA at a concentration of 10^6^/mL and incubated at 37 °C for 20 min in the cell culture incubator. ROS production in the cells was detected by flow cytometry on the FACSCalibur and analyzed by BD CellQuest Pro Software version 5.1.

### 2.5. Statistical Analysis

Data were presented as the mean ± standard deviation. Statistical significance was assessed using one-way analysis of variance (ANOVA), and the Tukey–Kramer test was used as a post-hoc test at a significance level of *p* < 0.05. The correlation analysis was carried out using Origin 2021 software (OriginLab, Northampton, MA, USA). All calculations and graphs were performed and constructed using GraphPad Prism 8.3.0 software (San Diego, CA, USA).

## 3. Results and Discussion

### 3.1. Physicochemical Properties of Polysaccharides from Different Dendrobium Species

#### 3.1.1. Chemical Compositions

Firstly, Table 1 lists the physicochemical properties of polysaccharides from different species of *Dendrobium*. The extraction rates of the polysaccharides vary widely, ranging from 1.47 ± 0.20% to 15.93 ± 1.07%. This discrepancy reflects the intrinsic characteristics of different *Dendrobium* species. Sevag reagent is commonly used for deproteinization of polysaccharides due to its mild reaction; however, this process inevitably leads to the loss of some protein-polysaccharide complexes, which might reduce the overall extraction rate of polysaccharides [23]. Therefore, the extraction rates shown in the table are lower than the actual content. Conversely, the highest extraction rate of 15.93 ± 1.07% was achieved by DHP. The next highest extraction rates were for DOP and DDP, which were greater than 8%. The extraction rates of DNP, DCP, DFP, and DAP were overall low and did not differ significantly (*p* > 0.05), ranging from 1.47 ± 0.20% to 3.54 ± 0.26%. The total content of the eight groups of *Dendrobium* polysaccharides ranged from 51.89 ± 6.91% to 80.57 ± 11.63%, and the protein content ranged from 1.3 ± 0.6% to 6.4 ± 3.9%, indicating that polysaccharides are the primary components of *Dendrobiums*. A higher polysaccharide extraction rate indicates that a significant proportion of target components can be obtained from the same unit mass of *Dendrobium*. This estimation will benefit the efficient utilization of *Dendrobium* and may also have a positive impact on its pharmacological effects.

Acetylation-modified polysaccharides can increase water solubility, thereby affecting the activity of polysaccharides [24,25]. Therefore, it is essential to determine the degree of acetylation of *Dendrobium* polysaccharides. According to Table 1, DOP has the highest degree of acetylation, followed by DDP and DHP. This finding suggested that these samples have high water solubility and altered bioactivity. Conversely, the other species of *Dendrobium* polysaccharides had lower levels of acetylation that did not differ significantly (*p* > 0.05). Previous studies have shown that *O*-acetyl-rich *Dendrobium* polysaccharides are beneficial to colon health [26], whereas deacetylation inactivates *Dendrobium* polysaccharides [27]. Specifically, the DAP sample had the lowest acetylation level, which might affect its water solubility and related biological functions. 

#### 3.1.2. Mw and PDI Data

**Figure 2:**
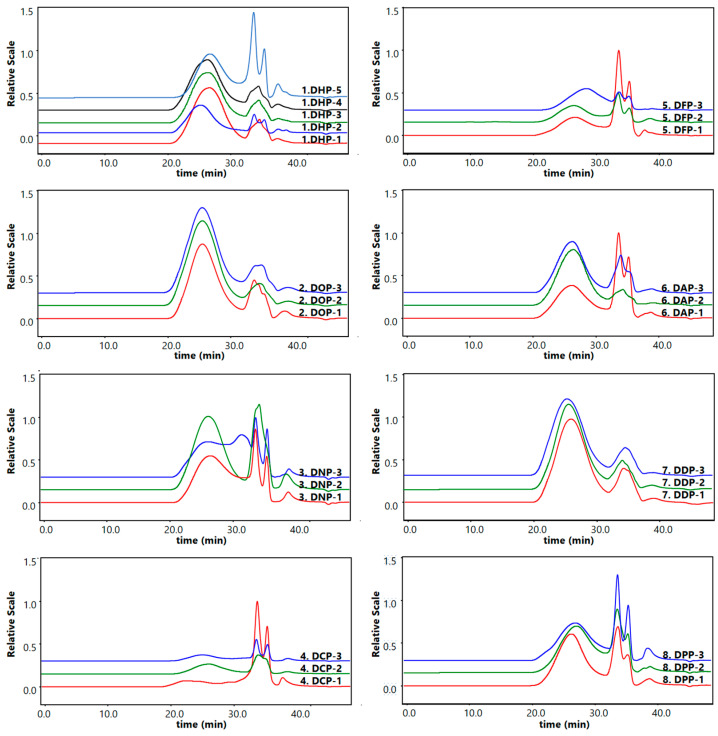
HPSEC-MALS-RID chromatograms of *Dendrobiums*.

The present study analyzed the molecular weights of different *Dendrobium* polysaccharides using HPSEC-MALS-RID (Figure 2). The Mw and PDI (Mw/Mn) of the eight *Dendrobium* polysaccharides are shown in Table 1. The Mw of the different *Dendrobium* polysaccharides was above 1 × 10^5^ Da, indicating the high molecular weights of these polysaccharides. The order of specific Mw is DHP < DFP < DNP < DOP < DAP < DCP < DDP < DPP. Among these, 8. DDP had the highest Mw, while no significant difference (*p* > 0.05) was observed between the Mw of the other species of *Dendrobium* polysaccharides. The PDI indicates the breadth of Mw distribution for polysaccharides. A low PDI value indicates a narrow Mw distribution, suggesting a high degree of uniformity in the polysaccharides [28]. Different *Dendrobium* polysaccharides showed a range of Mw distribution from the lowest 1.44 ± 0.05 to the highest 3.43 ± 0.78. Especially, DPP had the highest PDI value, indicating that its polysaccharides have a wide Mw distribution. Conversely, DHP had the lowest PDI value, indicating the most uniform Mw distribution of polysaccharides. 

The Mw range of all relevant studies on water-extracted *Dendrobium* polysaccharides is between 3 × 10^3^ and 1 × 10^7^ Da [29]. Generally, the bioactivity of polysaccharides is related to their Mw [30]. Another study showed varying antioxidant activity of different Mw components of *Dendrobium* polysaccharides [31]. Zhao et al. [32] showed that Mw is one of the key factors influencing the immunomodulatory activity of DOPs. These results indicate differences in Mw and molecular weight distribution of *Dendrobium* polysaccharides from different species, which might be related to the biological activities of the polysaccharides.

#### 3.1.3. Monosaccharide Composition

**Figure 3:**
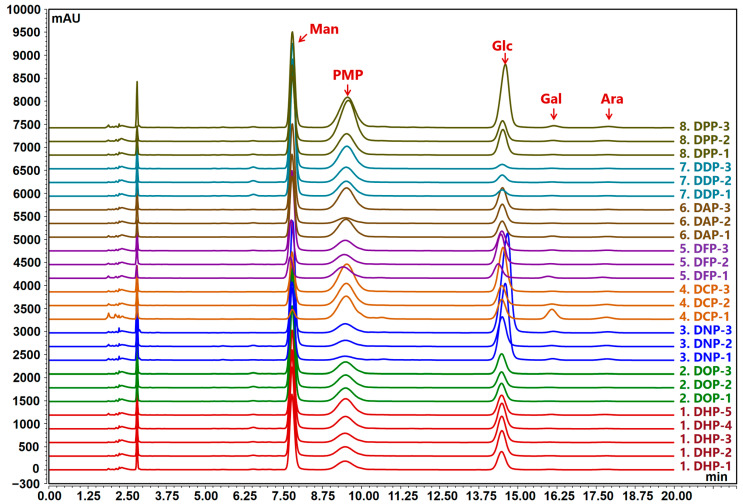
Chromatogram of the monosaccharide composition of *Dendrobium* polysaccharides.

Monosaccharides are the natural basic units that determine the unique structure and properties of polysaccharides. In this study, HPLC was used to determine the monosaccharide composition of different *Dendrobium* polysaccharides. The results show (Figure 3 and Table 1) that the samples of *Dendrobium* polysaccharides share similarities in their monosaccharide composition, primarily consisting of Man and Glc, along with minor amounts of Gal and Ara. However, the ratio of Man to Glc varies among the different *Dendrobium* polysaccharides. Among all the tested samples, DDP had the highest Man content, accounting for 93.86 ± 1.20% of the total composition, with the highest Man/Glc ratio of 12.86 ± 3.56. The content of Man as the primary monosaccharide component was the highest in DDP, followed by DOP, DAP, DHP, DPP, and DFP ranging from 52.62 ± 2.28% to 83.10 ± 2.43%, and the Man/Glc ratio was between 2.05 ± 0.20 and 3.41 ± 0.68. In contrast, DNP and DCP had high Glc content of 61.59 ± 7.70% and 54.02 ± 17.36%, and the Man/Glc ratio was 0.38 ± 0.13 and 0.59 ± 0.44, respectively.

These findings are consistent with those of a previous study on the polysaccharide composition of the *Dendrobium* genus, mainly consisting of Man and Glc, with small amounts of Gal [29]. Furthermore, according to the quality standards for *D. officinale* in the 2020 edition of the Chinese Pharmacopoeia, the specified Man/Glc peak area ratio is between 2.4 and 8.0, and fluctuations in this ratio are considered normal statistical variations. Lin [33] determined that the Man/Glc peak area ratio of 18 batches of *D. huoshanense* herbs was 0.36 to 3.23. This finding indicates that the natural fluctuation of the Man/Glc ratio is a normal phenomenon within the same species of *Dendrobium* polysaccharides. In summary, this information is crucial for understanding the structural and functional characteristics of *Dendrobium* polysaccharides that may serve as a reference for evaluating the quality and consistency of the herb.

### 3.2. Biological Experiments

#### 3.2.1. Construction of Models In Vitro for Preventing Oxidative Damage of GES-1 Cells

**Figure 4:**
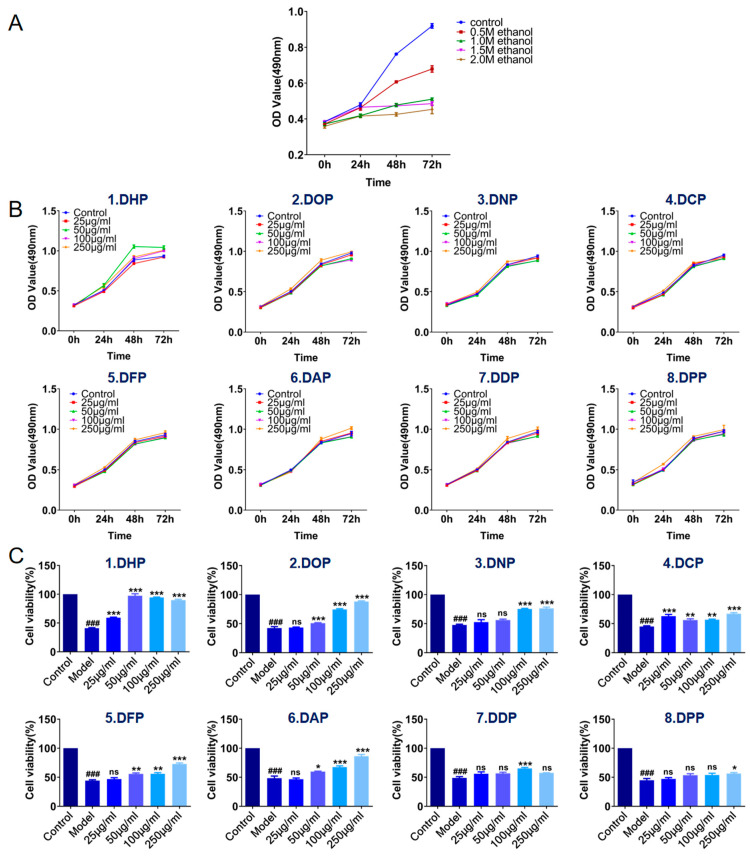
(**A**) Cell viability under ethanol treatment conditions. (**B**) Cell viability under treatment with different species of *Dendrobium* polysaccharides. (**C**) Effects of treatments with different species of *Dendrobium* polysaccharides on preventing ethanol damage in GES-1 cells in the model. Data are mean of three independent experiments (* *p* < 0.05, ** *p* < 0.01, *** *p* < 0.001 denote statistically significant differences between the treated and model groups; **^###^** *p* < 0.001 indicate a statistically significant difference between the control and model groups; ns, no significance).

Ethanol can cause cell dehydration, degeneration, and necrosis. Ethanol induction is a common method for modeling gastric mucosal injury. As shown in Figure 4A, the viability of ethanol-treated GES-1 cells decreased with increasing ethanol concentration after 48 h. The greater the ethanol concentration, the more serious the cell damage. When the cells were treated with 1.0 M ethanol for 48 h, their cell viability was approximately 50.35 ± 5.19% of that of the control, a significant difference (*p* < 0.01). Consecutively, the cell state was stable and met the requirements of this experiment. Therefore, the model group comprised ethanol (0.1 M)-damaged GES-1 cells.

Next, we determined whether *Dendrobium* polysaccharides exhibited cytotoxicity in GES-1 cells. Also, the most effective *Dendrobium* polysaccharides treatment time and concentration were screened to establish an in vitro prevention model for GES-1 cells (Figure 4B). Cell proliferation was enhanced after intervening normal GES-1 cells with eight groups of different species of *Dendrobium* polysaccharides (25–250 μg/mL) for 24 h. This phenomenon indicated that 25–250 μg/mL concentrations of *Dendrobium* polysaccharides are non-toxic to GES-1 cells and promote cell growth. Therefore, *Dendrobium* polysaccharide can be used as an intervention in damaged GES-1 cells. DHP was more effective than other *Dendrobium* polysaccharides when added to the cells at a lower concentration of 50 μg/mL for 48-h potency.

After *Dendrobium* polysaccharide treatment for 48 h, the GES-1 cells were exposed to 1.0 M ethanol for 4 h. Consequently, the cell survival rate of the model group was 45.34%, which differed significantly (*p* < 0.001) compared to that of the control group, indicating successful modeling. Figure 4C shows the concentration dependence of the polysaccharide treatment groups of DOP, DNP, DFP, DAP, and DPP, whereas DHP, DCP, and DDP decreased cell viability at a specific concentration. This phenomenon may be attributed to the saturation effect at the plateau phase. Overall, the protective effect of the low concentration of 50 μg/mL DHP polysaccharide treatment group was similar to that of the control group (*p* > 0.05), and the cell viability could reach 97.32% of the control group.

In conclusion, all the *Dendrobium* polysaccharides from different groups exerted a protective effect against ethanol-induced GES-1 cell damage. Nonetheless, based on the polysaccharide composition, Dendrobium huoshanense was slightly better than other varieties in the protective effect of GES-1 cells.

#### 3.2.2. Effects of *Dendrobium* Polysaccharides on Scratch Repair in GES-1 Cells

**Figure 5:**
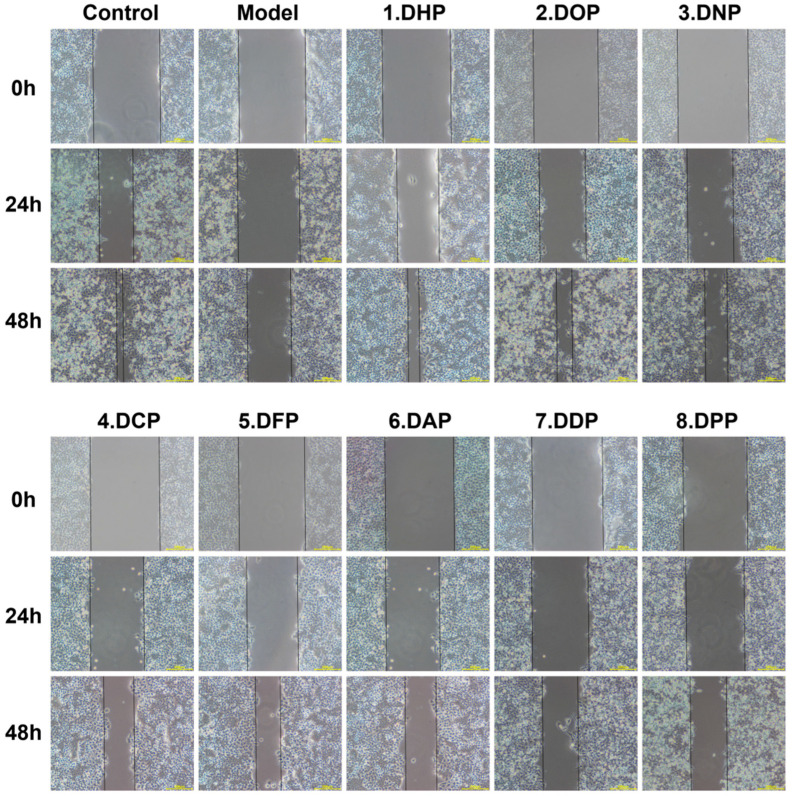

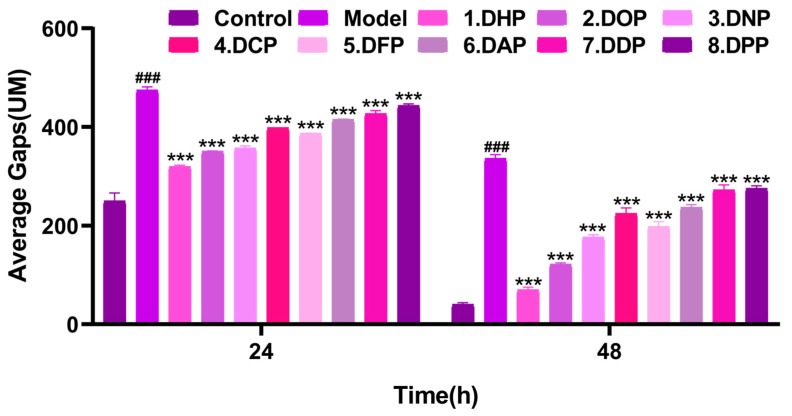
Comparison of the repair effects of different *Dendrobium* polysaccharides after GES-1 injury. Data are the mean of three independent experiments (*** *p* < 0.001 denote statistically significant differences between the treated and model groups; **^###^** *p* < 0.001 denotes a statistically significant difference between the control and model groups).

The proliferation and migration ability of GES-1 cells are critical factors affecting the healing of mucosal injuries [34,35]. Thus, promoting cell proliferation and migration to the site of mucosal injury is the key to improving mucosal protection. Figure 5 shows that different species of *Dendrobium* polysaccharides can promote the migration and growth of GES-1 cells to the damaged area (i.e., reduce the cell average gaps) to different degrees. After incubating the damaged GES-1 cells with eight different *Dendrobium* polysaccharides, each at 50 μg/mL for 24 h, the repair rates of the damaged areas were 13.53–38.10%. Among these, DHP and DOP showed the best repair performance with 38.08 ± 3.12% and 33.33 ± 1.10%, respectively, which was significantly higher than that of the model group with 4.77 ± 2.56% (*p* < 0.001). The repair effect of *Dendrobium* polysaccharide was more obvious after 48-h damage of GES-1 cells, but the gap between the repair rates of eight different *Dendrobium* polysaccharides increased gradually (35.28–83.13%). Among these polysaccharides, DHP had the best repair effect (83.13 ± 1.05%), followed by DOP with a high repair rate (72.26 ± 2.65%); these values were significantly higher than that of the model group (46.51 ± 6.22%) (*p* < 0.001). In conclusion, different species of *Dendrobium* polysaccharides showed different degrees of migration and repair effects on GES-1-damaged cells, among which DHP had the better effect.

#### 3.2.3. SOD, CAT, and GSH-px Activity Measurements and MDA Content Assay

**Figure 6:**
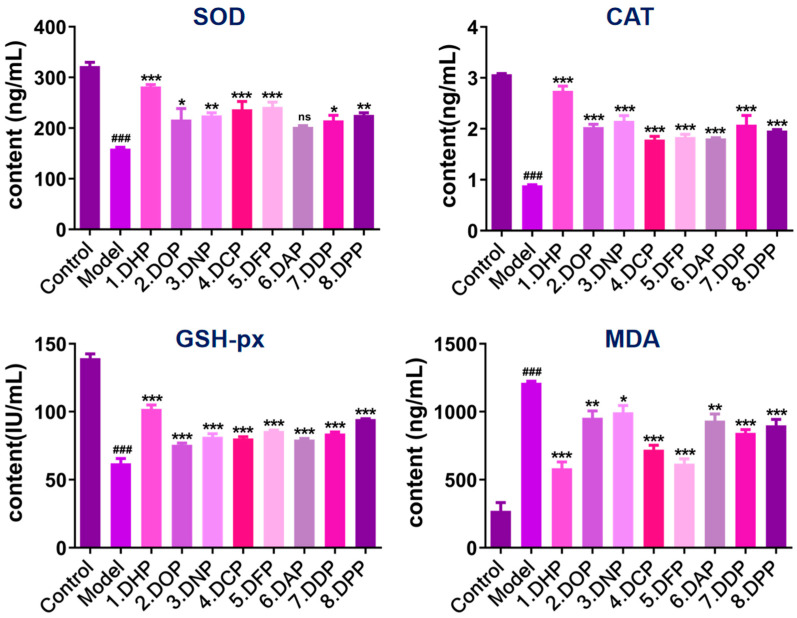
Intracellular levels of SOD, CAT, GSH-Px, and MDA. (* *p* < 0.05, ** *p* < 0.01, *** *p* < 0.001 denote statistically significant differences between the treated and model groups; **^###^** *p* < 0.001 denotes a statistically significant difference between the control and model groups; ns, no significance).

Oxidative stress is one of the major factors in gastric mucosal injury, and antioxidant enzymes play a crucial role in gastric mucosal homeostatic balance [36]. Antioxidant enzymes, such as SOD, CAT, and GSH-px, eliminate excess ROS to protect gastric mucosal cells from oxidative stress damage [37]. Overall, the activities of antioxidant enzymes SOD, CAT, and GSH-px were significantly reduced (*p* < 0.001) in the injury model group compared to the control group (Figure 6). GES-1 cells pretreated with different groups of *Dendrobium* polysaccharides showed a variable increase in SOD, CAT, and GSH-px activities. The DHP pretreatment group had the better recovery effect on SOD and CAT activity, with no statistically significant difference compared to the control group (*p* > 0.05), and improved the activity of GSH-px. These results showed that different groups of *Dendrobium* polysaccharide pretreatment can effectively increase the activities of SOD, CAT, and GSH-px and inhibit the ethanol-induced oxidative stress damage to cells.

MDA is a lipid peroxidation product, a vital marker of oxidative damage [38]. The ethanol-induced injury increased the MDA content and lipid peroxidation reaction in GES-1 cells [39]. Compared to the control group, the MDA level in the model group was increased significantly (*p* < 0.001). The different groups of *Dendrobium* polysaccharides-pretreated GES-1 cells reduced the MDA content to varying degrees. DHP, DCP, DPP, DDP, and DPP showed extremely significant differences compared to the model group (*p* < 0.001) under the same effective concentration (50 μg/mL). DOP and DAP have similar effects (*p* < 0.01), with a highly significant difference, whereas DNP had the lowest significance compared to the model group (*p* < 0.05). These results indicated that polysaccharides from different species of *Dendrobium* can alleviate ethanol-induced lipid oxidative damage in GES-1 cells. DHP had the most significant effect among these polysaccharides, with a decrease in MDA content of about 48.14% compared to the model group. Overall, DHP pretreatment alleviates ethanol-induced lipid oxidative damage in GES-1 cells.

#### 3.2.4. Effects of *Dendrobium* Polysaccharides on ROS Level in GES-1 Cells

**Figure 7:**
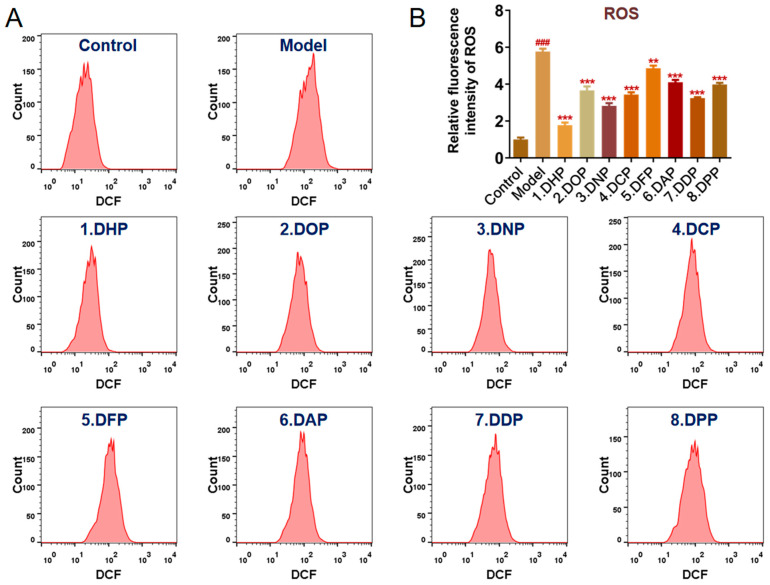
Effect of treatment with different species of *Dendrobium* polysaccharides on ROS levels in ethanol-induced GES-1 cells. (**A**) DCF fluorescence spectrum of *Dendrobium* polysaccharides. (**B**) Relative fluorescence intensity of ROS. (** *p* < 0.01, *** *p* < 0.001 denote statistically significant differences between the treated and model groups; ^###^ *p* < 0.001 denotes a statistically significant difference between the control and model groups).

ROS are involved in the mechanism of cellular oxidative stress, and inflammatory factors released by damaged cells can promote the production and accumulation of ROS [40]. In the present study, ROS levels were used as oxidative stress markers. Flow cytometry was used to assess ROS levels in GES-1 cells treated with different species of *Dendrobium* polysaccharides (Figure 7). The horizontal axis represents the DCFH-DA fluorescence signal value, i.e., the fluorescence intensity of the detected DCF. Compared to the control, the fluorescence peak shifted when GES-1 cells were treated with ethanol. These shifts of the fluorescence peaks in each group were alleviated to different degrees after pretreatment with *Dendrobium* polysaccharides. Compared to the control group, the ROS level in the model group increased by about 5.7 times, indicating that ROS is produced in GES-1 cells in the presence of ethanol. Different *Dendrobium* polysaccharides exhibited varying degrees of intracellular ROS scavenging effects. Moreover, the ROS levels were decreased significantly in all pretreatment groups compared to the ethanol-induced model group (*p* < 0.01). Only the ROS level of DHP was closest to that of the control group, indicating that the DHP group is the most effective in eliminating intracellularly generated ROS. These results suggested that DHP attenuates cellular ROS accumulation and exerts a protective effect on ethanol-induced oxidative stress in gastric mucosal cells.

## 4. Discussion

All eight *Dendrobium* polysaccharides mainly consist of Man and Glc, with a small amount of Gal and Ara; however, the proportion of monosaccharides is varied. The molecular weights of all the *Dendrobium* polysaccharides are >1 × 10^5^ Da, among which DHP has the smallest molecular weight. Additionally, *Dendrobium* polysaccharides alleviate ethanol-induced oxidative stress and inflammatory response in gastric mucosal cells by enhancing the activity of antioxidant enzymes (SOD, GSH-PX, and CAT) and reducing the levels of MDA and ROS. The components also promote cell migration and repair, protecting the gastric mucosa from damage. Based on the polysaccharide composition, although all *Dendrobium* had protective effects on gastric mucosa, DHP showed better protective effects in general. Pan et al. [41] compared the hypoglycemic and antioxidant activities of four *Dendrobium* polysaccharides (DHP, DOP, DNP, DCP) and found that DHP was the most effective. However, this difference in activity was only presumed to be the difference between the physicochemical properties of the polysaccharides, and the correlation between activity and structure was not elucidated in detail. In contrast, Liu et al. [42] found that the anti-gastric cancer activity of DHP was closely related to its Mw and *O*-acetyl groups.

**Figure 8:**
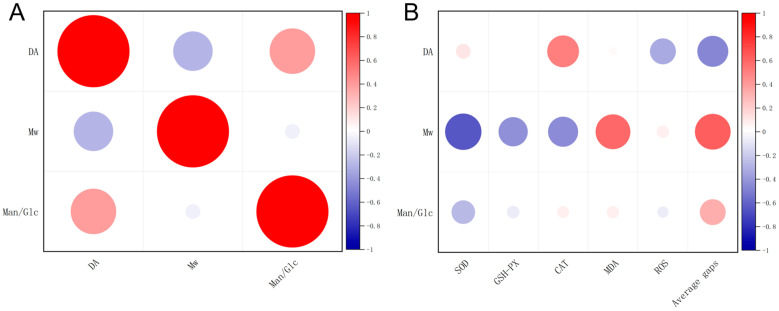
Correlation matrix analysis. (**A**) Structure; (**B**) correlation plot between structural characters and gastric mucosal protection.

In order to further investigate the structure-activity relationship of *Dendrobium* polysaccharides in gastric mucosal protection, we further carried out a correlation analysis. First, Figure 8A corresponds to the structural composition of *Dendrobium* polysaccharides. A positive correlation was established between M/G and DA with a Pearson’s correlation coefficient of r = 0.40, indicating that an increased M/G ratio is associated with an increased degree of acetylation in the polysaccharides. Concurrently, a negative correlation was observed between Mw and DA (Pearson’s correlation coefficient of r = −0.30), suggesting that polysaccharides with higher molecular weights tend to have lower degrees of acetylation.

Figure 8B elucidates the influence of Mw, DA, and M/G on various bioactivity indices. Interestingly, a significant correlation was established between DA and CAT, ROS, and the average gaps of cells (Pearson’s correlation coefficients were r = 0.50, r = −0.33, and r = −0.48, respectively). This finding suggests that an elevated DA may be conducive to increased CAT activity, decreased ROS production, and reduced cell average gaps; these phenomena are indicators of enhanced cellular proliferation and migration. In addition, Mw is correlated with SOD, CAT, GSH-Px, MDA, and average gaps (Pearson’s correlation coefficients were r = −0.65, r = −0.43, r = −0.45, r = 0.60, and r = 0.63, respectively). These data suggested that polysaccharides with low Mw may enhance the activity of antioxidant enzymes, decrease lipid peroxidation product MDA levels, and promote cellular proliferation and migration. The M/G ratio is weakly correlated with SOD and average gaps, with correlation coefficients of r = −0.28 and r = 0.33; these values indicate that variations in M/G have a lesser pronounced effect on these parameters compared to other factors.

In summary, the Mw of polysaccharides has the most significant impact on biochemical indicators related to gastric mucosal protective effects, followed by DA, while the M/G ratio does not exhibit significant correlations. Overall, DOP is second only to DHP in terms of gastric mucosal protection. Although the composition of DOP and DHP monosaccharides is similar, the ratio is different. Moreover, DOP has the highest DA value, with a slightly larger Mw. Large Mw makes it difficult for organisms to absorb DOP, and small Mw causes loss of the activity of the monosaccharide [43,44]. Secondly, high DA values can destabilize the complex molecular structure, resulting in reduced or loss of biological activity and function [45]. Thus, DA and Mw may affect the biological activity of *Dendrobium* polysaccharides.

## 5. Conclusions

In summary, eight different *Dendrobium* polysaccharides were extracted and characterized in this study, and their gastric mucosal protective effects were compared. All the *Dendrobium* polysaccharides involved in the comparison have been proven to alleviate the harmful effects of ethanol on the acute gastric mucosal injury model induced by GES-1 cells to varying degrees, through enhancing antioxidant capacity and suppressing oxidative stress. Among them, the cell viability of DHP (50 μg/mL) was 97.32% of that of the control group, and the cell repair effect was the better (83.13 ± 1.05%), and its antioxidant capacity was the strongest. Overall, DHP has the greatest potential for gastric mucosal protection. These phenomena could be attributed to the smallest Mw and DA that prompt the manifestation of the biological activities of the polysaccharides. Taken together, these findings provide an in-depth understanding of the chemical structure and bioactivity of *Dendrobium* polysaccharides and theoretical support for their practical application as gastric mucosa-protecting components in dietary supplements and functional foods. Although the present study has provided a comprehensive and systematic assessment at the cellular level, future studies need to be further confirmed by animal model experiments.

## Figures and Tables

**Table 1:** Physicochemical properties of *Dendrobiums* (*n* indicates batch). Values are mean ± standard deviation (SD), superscripts a–d indicate significant differences (*p* < 0.05) among various species of *Dendrobium* polysaccharides; statistical significance was analyzed by ANOVA and Duncan’s test.

Parameter	1. DHP (*n* = 5)	2. DOP (*n* = 3)	3. DNP (*n* = 3)	4. DCP (*n* = 3)	5. DFP (*n* = 3)	6. DAP (*n* = 3)	7. DDP (*n* =3)	8. DPP (*n* =3)
Polysaccharide yields (%)	15.93 ± 1.07 ^a^	8.46 ± 0.62 ^b^	2.53 ± 0.36 ^c^	1.47 ± 0.20 ^c^	1.76 ± 0.10 ^c^	3.54 ± 0.26 ^c^	10.12 ± 0.68 ^a^	5.20 ± 0.689 ^b^
Total polysaccharides (%)	65.88 ± 4.30 ^b^	58.00 ± 3.01^b^	63.20 ± 13.07 ^b^	51.89 ± 6.91 ^c^	72.40 ± 13.29 ^a^	80.57 ± 11.63 ^a^	66.46 ± 3.87 ^b^	57.71 ± 4.50 ^b^
Protein content (%)	1.62 ± 1.10 ^c^	1.25 ± 0.62 ^c^	4.40 ± 1.72 ^b^	6.43 ± 3.88 ^a^	5.83 ± 3.61 ^a^	3.30 ± 1.71 ^b^	1.86 ± 0.31 ^c^	3.12 ± 1.72 ^b^
Acetylation degree	0.33 ± 0.03 ^b^	0.48 ± 0.03 ^a^	0.28 ± 0.10 ^b^	0.26 ± 0.11 ^b^	0.23 ± 0.07 ^b^	0.17 ± 0.03 ^c^	0.37 ± 0.04 ^a^	0.23 ± 0.07 ^b^
Mw (×10^5^ Da)	1.31 ± 0.17 ^c^	1.82 ± 0.34 ^c^	1.80 ± 0.04 ^c^	2.14 ± 0.24 ^b^	1.70 ± 0.06 ^c^	1.91 ± 0.27 ^c^	2.24 ± 0.31 ^b^	5.84 ± 2.49 ^a^
PDI	1.50 ± 0.14 ^c^	1.44 ± 0.05 ^c^	1.84 ± 0.17 ^b^	1.75 ± 0.15 ^b^	1.63 ± 0.04 ^c^	1.75 ± 0.36 ^b^	1.47 ± 0.09 ^c^	3.43 ± 0.78 ^a^
Man (%)	74.95 ± 1.94 ^b^	83.10 ± 2.43 ^a^	35.76 ± 7.45 ^d^	42.51 ± 14.82 ^c^	52.62 ± 2.28 ^c^	75.17 ± 1.35 ^a^	93.86 ± 1.20 ^a^	66.88 ± 10.52 ^b^
Glc (%)	23.32 ± 1.64 ^b^	15.81 ± 2.44 ^c^	61.59 ± 7.70 ^a^	54.02 ± 17.36 ^a^	41.74 ± 3.51^a^	22.59 ± 0.98 ^b^	4.86 ± 1.24 ^c^	30.08 ± 9.95 ^b^
Gal (%)	0.94 ± 0.34 ^c^	0.66 ± 0.05 ^c^	1.49 ± 0.25 ^b^	2.20 ± 1.87 ^b^	3.68 ± 3.36 ^a^	1.28 ± 1.00 ^b^	0.73 ± 0.03 ^c^	1.63 ± 0.67 ^b^
Ara (%)	0.79 ± 0.46 ^c^	0.43 ± 0.08 ^c^	1.15 ± 0.40 ^b^	1.26 ± 0.68 ^b^	1.97 ± 1.82 ^a^	0.96 ± 0.52 ^c^	0.55 ± 0.04 ^c^	1.41 ± 0.59 ^b^
Man/Glc	2.05 ± 0.20 ^b^	3.41 ± 0.68 ^b^	0.38 ± 0.13 ^d^	0.59 ± 0.44 ^c^	0.80 ± 0.06 ^c^	2.12 ± 0.10 ^b^	12.86 ± 3.56 ^a^	1.55 ± 0.62 ^b^

## Data Availability

The original contributions presented in the study are included in the article, further inquiries can be directed to the corresponding authors.

## Abbreviations

Glc: glucose; Gal, galactose; Ara, arabinose; MDA, Malondialdehyde; ROS, reactive oxygen species; SOD, Superoxide Dismutase; GSH-Px, Glutathione Peroxidase; CAT, Catalase; DHP, *Dendrobium huoshanense* polysaccharides; DOP, *Dendrobium officinale* polysaccharides; DNP, *Dendrobium nobile* polysaccharides; DCP, *Dendrobium chrysotoxum* polysaccharides; DFP, *Dendrobium fimbriatum* polysaccharides; DAP, *Dendrobium aphyllum* polysaccharides; DDP, *Dendrobium devonianum* polysaccharides; DPP, *Dendrobium pierardii* polysaccharides; PMP, Phenyl-3-methyl-5-pyrazolone; TFA, Trifluoroacetic acid; SDS-PAGE, sodium dodecyl sulfate-polyacrylamide gel electrophoresis; Anti-GAPDH, glyceraldehyde-3-phosphate dehydrogenase); HPLC, high-performance liquid chromatography; Mw, molecular weight; PDI, polymer dispersity index; HPSEC-MALS-RID, High-performance size exclusion chromatography—Multi angle light scattering—Refractive index detector; DA, acetylation; PBS, phosphate-buffered saline; DCFH-DA, 2,7-dichlorofluorescein-diacetate.
